# Vitamin D Supplementation for Childhood Asthma: A Systematic Review and Meta-Analysis

**DOI:** 10.1371/journal.pone.0136841

**Published:** 2015-08-31

**Authors:** Bruno D. Riverin, Jonathon L. Maguire, Patricia Li

**Affiliations:** 1 Department of Pediatrics, Montreal Children's Hospital, McGill University Health Centre, Montreal, Quebec, Canada; 2 Department of Epidemiology, Biostatistics & Occupational Health, McGill University, Montreal, Quebec, Canada; 3 The Applied Health Research Centre of the Li Ka Shing Knowledge Institute of St. Michael’s Hospital, University of Toronto, Toronto, Ontario, Canada; 4 Department of Pediatrics, St. Michael’s Hospital, Toronto, Ontario, Canada; 5 Pediatric Outcomes Research Team (PORT), Division of Pediatric Medicine, Department of Pediatrics, University of Toronto, The Hospital for Sick Children, Toronto, Ontario, Canada; Telethon Institute for Child Health Research, AUSTRALIA

## Abstract

**Importance:**

There is growing evidence that vitamin D plays a role in the pathogenesis of asthma but it is unclear whether supplementation during childhood may improve asthma outcomes.

**Objectives:**

The objective of this systematic review and meta-analysis was to evaluate the efficacy and safety of vitamin D supplementation as a treatment or adjunct treatment for asthma.

**Data Sources:**

We searched MEDLINE, Embase, CENTRAL, and CINAHL through July 2014.

**Study Selection:**

We included RCTs that evaluated vitamin D supplementation in children versus active control or placebo for asthma.

**Data Extraction and Synthesis:**

One reviewer extracted data and one reviewer verified data accuracy. We qualitatively summarized the main results of efficacy and safety and meta-analyzed data on comparable outcomes across studies. We used GRADE for strength of evidence.

**Main Outcome Measures:**

Main planned outcomes measures were ED visits and hospitalizations. As secondary outcomes, we examined measures of asthma control, including frequency of asthma exacerbations, asthma symptom scores, measures of lung function, β_2_-agonist use and daily steroid use, adverse events and 25-hydroxyvitamin D levels.

**Results:**

Eight RCTs (one parallel, one crossover design) comprising 573 children aged 3 to 18 years were included. One study (moderate-quality, n = 100) reported significantly less ED visits for children treated with vitamin D. No other studies examined the primary outcome (ED visits and hospitalizations). There was a reduced risk of asthma exacerbations in children receiving vitamin D (low-quality; RR 0.41, 95% CI 0.27 to 0.63, 3 studies, n = 378). There was no significant effect for asthma symptom scores and lung function. The serum 25(OH)D level was higher in the vitamin D group at the end of the intervention (low-quality; MD 19.66 nmol/L, 95% CI 5.96 nmol/L to 33.37 nmol/L, 5 studies, n = 167).

**Limitations:**

We identified a high degree of clinical diversity (interventions and outcomes) and methodological heterogeneity (sample size and risk of bias) in included trials.

**Conclusions and Relevance:**

Randomized controlled trials provide some low-quality evidence to support vitamin D supplementation for the reduction of asthma exacerbations. Evidence on the benefits of vitamin D supplementation for other asthma-related outcomes in children is either limited or inconclusive. We recommend that future trials focus on patient-relevant outcomes that are comparable across studies, including standardized definitions of asthma exacerbations.

## Introduction

There is growing evidence that suggests a relationship between vitamin D and asthma. Based on results from epidemiologic and animal studies, vitamin D may play a role in the pathogenesis of asthma via its effects on the innate and adaptive immune system. [1–7] Among children with asthma, several cross-sectional studies have reported associations between low blood concentrations of vitamin D and increased severity of disease. [8–12]

Formal guidelines on the vitamin D requirements for the healthy population have largely focused on evidence for skeletal manifestations of vitamin D deficiency. [13] There is uncertainty as to whether vitamin D supplementation during childhood may improve asthma outcomes. Given the ongoing research and emerging evidence on this topic, it is important to synthesize the evidence for this intervention to support decision-making for patients and for health care providers.

### Objective

We sought to systematically search and critically assess the evidence from clinical trials on the efficacy and safety of vitamin D supplementation as a treatment or an adjunct to other asthma treatments to improve asthma outcomes, including ED visits and hospital admissions, measures of asthma control, and 25-hydroxyvitamin D levels in children.

## Methods

### Data Sources

We identified references by searching MEDLINE via the Ovid interface, Embase, CINAHL and the Cochrane Central Register of Controlled Trials (CENTRAL) since inception through July 2014 using regular search terms related to vitamin D and asthma and Medical Subject Headings (MeSH) search terms in MEDLINE. Regular search terms used were: vitamin D, cholecalciferol, calcitriol, ergocalciferol, 25-hydroxyvitamin D_2_, child, infant, preschool, adolescent, and asthma. MeSH search terms used were: “Vitamin D OR 25-hydroxyvitamin D_2_ OR Cholecalciferol OR Ergocalciferol” AND “Child OR Infant OR Preschool OR Adolescent” AND “Asthma”. We manually searched the reference lists of published reviews and meta-analyses for additional studies. Full search strategies are listed in the Supporting Information (see S1 Table).

### Study Selection Criteria

We considered randomized controlled trials (RCTs) with parallel or cross-over design that evaluated the effect of vitamin D supplementation alone or as an adjunct to other forms of asthma treatment versus active control or placebo in children aged 0 to 18 years with asthma. Studies met the following criteria: (1) participants: children with diagnosed asthma (doctor’s diagnosis and/or objective criteria); (2) interventions: all preparations of vitamin D as oral supplement, at any dose and for any duration, including preparations containing supplemental vitamin D; (3) comparisons: compared to placebo, no supplementation or standard care; (4) outcomes: at least one of the primary or secondary outcomes examined in the current review. The primary outcome was ED visits and/or hospital admissions for asthma exacerbations and secondary outcomes included frequency of asthma exacerbations, asthma severity or symptom scores, lung function (forced expiratory volume in one second [FEV1], peak expiratory flow [PEF]), β_2_-agonist use, daily steroid use, days lost from work/school, nocturnal awakening, serum 25-hydroxyvitamin D concentration, withdrawal from trial due to adverse events, and adverse events (such as vitamin D toxicity). Eligible trials investigating the efficacy and/or safety of vitamin D as an adjunct to other forms of asthma treatment were also included. We excluded studies that looked at maternal vitamin D supplementation during pregnancy or lactation or those looking at fish oil supplementation without information on vitamin D dosage.

### Study Selection

Two investigators (BR, PL) independently scanned the abstract of every retrieved record for eligibility. The same two reviewers investigated those eligible for inclusion further as full text.

### Data Extraction and Assessment of Risk of Bias in Included Studies

For studies that satisfied the inclusion criteria, one reviewer (BR) independently extracted data using a standardized form developed *a priori*. We collected information on characteristics of study populations, interventions, and outcomes. We contacted the authors of the trials included in the review to obtain additional data on outcome measures that would allow for a meta-analysis of results across trials. A second reviewer (PL) verified and confirmed the extracted data. Two reviewers independently assessed the quality of evidence and risk of bias in the included studies according to the GRADE guidelines. [14–20]

### Data Synthesis

Quantitative data from studies that reported on comparable outcome measures were combined by conducting a meta-analysis using the change in outcomes from baseline or outcomes reported at the end of the study intervention period. For continuous outcomes, the weighted mean difference (WMD) between the intervention and control group were used as measures of effect size. Weights were assigned based on the inverse variances of the effect size estimates. If studies reported continuous outcomes as different units, we calculated the standardized mean difference (SMD) between the two groups. We combined data from the included studies using a random effects model. Random effects models are more conservative and account for variability both within and between included studies. For dichotomous outcomes the random effect model was used to estimate the pooled relative risk (RR) and 95% confidence interval (CI).

We examined statistical heterogeneity between results of different studies by the overlap of their confidence interval (95% CI). We estimated heterogeneity where applicable by the Cochrane Q (Chi-square test) and the *I* ^*2*^ statistic. We performed sensitivity analysis using both fixed and random effects meta-analyses to assess the robustness of the results to the method used. Data was meta-analyzed using Review Manager, version 5 (RevMan, The Cochrane Collaboration, Oxford, United Kingdom). This review follows the recommendation of the PRISMA statement (see S2 Table). [21]

## Results

### Study selection

**Fig 1:**
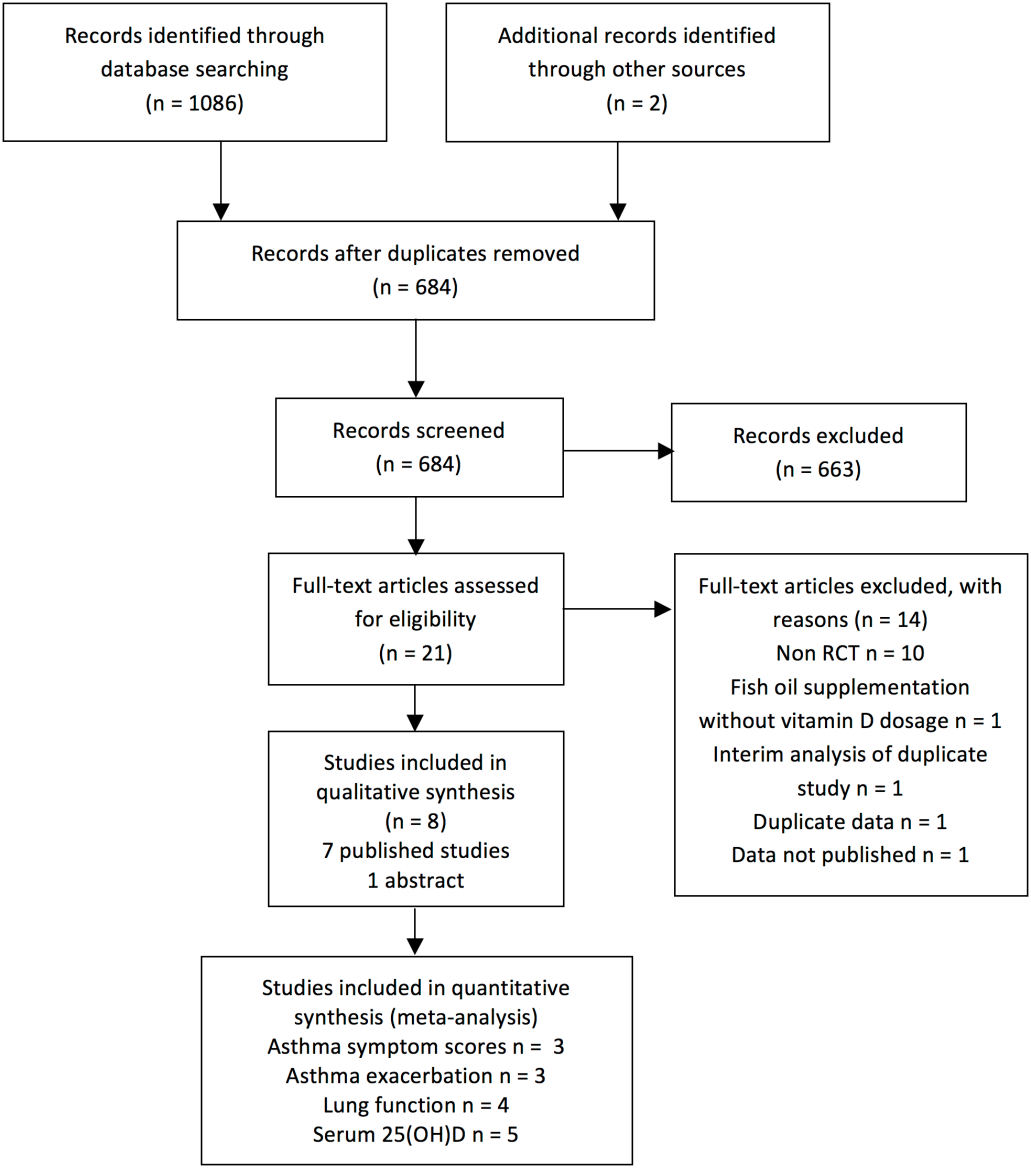
PRISMA flowchart.

We identified 1086 eligible citations from the four databases searched (see Fig 1). There were 402 duplicate citations and an additional 663 records did not meet our inclusion criteria after careful title and abstract review. Of the 21 articles included for full-text review, [22–41] exclusion was ascertained for publications with observational designs. [22,23,25,26,30,33,35,37,38] Two citations were found to be duplicates of studies published at a later date, and one citation was excluded due to incomplete reporting, [41] resulting in 8 included trials.

### Characteristics of included studies

Table 1 describes the eight trials included in the review. Seven randomized controlled parallel-group trials [24, 27, 31, 34, 36, 42, 43] and one randomized controlled crossover trial [39] comprising 573 asthmatic children aged 3 to 18 years were included (Table 1). Asthma related outcome measures were the primary focus of six studies, [24,27,31,36,42,43] whereas the primary outcomes of two studies were growth and bone turnover [39] and seasonal influenza A. [34] Children included in these studies were 1) previously diagnosed with asthma by a physician in four studies, [27,34,42,43] 2) newly diagnosed patients with asthma in two studies, [24,31] or 3) had a disease duration of one year or more. [36,39] Sample sizes ranged from 17 (crossover trial) to 430 participants. Trials recruited from subspecialty allergy clinics, hospitals and university health centers and private practices. All included trials were conducted at latitudes between the 28^th^ and the 55^th^ parallel north. The duration of the interventions ranged from 1 month to 12 months and doses ranged from 1000 IU/week to 60000 IU/month (weighted mean 1058 IU/d; range 143 IU/d to 2000IU/d). Five trials investigated the effect of vitamin D supplementation as an adjunct therapy to other forms of asthma treatment, including inhaled steroids, [24,31,39] oral steroids and specific immunotherapy (SIT) for house dust mite, [36] and SIT alone. [43] In one trial, participants received vitamin A and the B vitamins as constituents of the vitamin D3-containing tablet. [39] Compliance with the vitamin D supplementation intervention was reported in only two trials; one trial reported that one patient in each treatment arm were excluded from the study because of noncompliance with the intervention, [43] and another reported that 96% of participants analyzed complied with the intervention based on logs provided at each visit. [34]

**Table 1:** Characteristics of included studies.

Study			Population				Intervention					
Source	RCT Design	Country (Latitude)	Age range (years)	N	Recruitment setting	Diagnosis of asthma	Type	Regimen	Duration in months (start-end season)	Co-intervention	Outcomes measured	Baseline and follow-up assessments (months)
Baris at al 2014 [43]	Parallel	Turkey (40°N)	5 to 15	50^a^	Subspecialty medical clinic	Medical history, physical examination findings, postbronchodilator changes in FEV1	D_3_	650 IU/d	12 (w—w)	SCIT	AE, AS, ICS, LF, VD, AdvE	-1, 0, 6, 12
Yadav et al 2013 [42]	Parallel	India (29°N)	3 to 14	100	Subspecialty medical clinic	Physician diagnosis by GINA guidelines	D_3_	60 000 IU/mo	6 (sp—w)	None	ED, AE, AS, ICS, LF	Monthly
Darabi et al 2013 [24]	Parallel	Iran (32°N)	6 to 14	80	Subspecialty medical clinic	Newly diagnosed asthma	D_3_	500 IU/d	6 (w/sp—sp/su)	Fluticasone 500 μg/d	AE, AS, LF VD	NR
Lewis et al 2012 [27]	Parallel	United States (41°N)	6 to 17	30	University medical center	By physician^d^	D_3_	1 000 IU/d	12 (w—w)	None	AS, LF, VD	0, 6, 12
Majak et al 2011 [31]	Parallel	Poland (52°N)	5 to 18	48	Subspecialty medical clinic	Newly diagnosed asthma; sensitive only to house dust mites	D_3_	500 IU/d	6 (f—su)	Budesonide 800 mg/d	AE, AS, LF VD	0, 2, 4, 6
Urashima et al 2010 [34]	Multicentric Parallel	Japan (36°N)	6 to 15	430^b^	Hospitals and private practices	By physician	D_3_	1 200 IU/d	4 (w- sp)	None	AE, AdvE	0, 4
Majak et al 2009 [36]	Parallel	Poland (52°N)	6 to 12	54^c^	Subspecialty medical clinic	IgE-dependent asthma with regular symptoms; disease duration ≥2 years	D_3_	1 000 IU/week in a single dose	12 (sp—su)	Prednisone (20mg) + SIT	AS, ICS, LF, VD	7 months pre-study, 0, 3, 12
Schou et al 2003 [39]	Crossover	Denmark (55°N)	6 to 14	17	NR	Diagnosis by international guidelines^‡^; treated with glucocorticoid 1 year before study entry	D_3_	600 IU/d	1 (w—w))	Budesonide, Turbulhaler, vitamin A, B	AS, ICS, LF, β_2_, VD	0, 1

Abbreviations: w, winter; sp, spring; su, summer; f, fall; NR, not reported; HDM, house dust mites; SIT, specific immunotherapy; ED, emergency department visits; AE, asthma exacerbations; AS, asthma severity/symptoms; ICS, inhaled corticosteroid use; β_2_, β_2_ agonist use; LF, lung function; VD, serum 25(OH)D concentration; AdvE, adverse events.

^a^55 participants were randomized into three groups: pharmacotherapy group, SIT group, SIT + vitamin D group. Only SIT (n = 15) and SIT + vitamin D (n = 17) were included in this review (n = 32).

^b^430 participants were randomized into vitamin D or placebo group. Only those who were previously diagnosed with asthma were used in the calculation of the RR presented in the meta-analysis (n = 230).

^c^54 participants were randomized into three groups: placebo group, steroid group, steroid + vitamin D group. Only steroid group (n = 18) and steroid + vitamin D group (n = 18) were used (n = 36).

^d^Information obtained directly from authors.

### Risk of bias (study limitations)

We summarized the assessment of the study limitations of each included trials consistent with the GRADE guidelines [15] in a supporting information file (S3 Table). It was unclear in three trials if the allocation to the treatment group was adequately concealed. [24,27,36] It was unclear in four trials if both patients and outcome assessors were blinded to the treatment arm to which patients were allocated. [24,27,31,39] Three trials lost greater than 20% of participants to follow-up. [24,27,34] Selective outcome reporting could have occurred, as we did not have access to a protocol developed *à priori* for any of the eight trials. [44] One study used a crossover design with an insufficient wash-out period of two weeks (serum 25(OH)D has a half-life of three to four weeks). We could not ascertain that the outcomes were valid in one study because of unclear reporting of outcome measurement. [42]

### Outcomes

Table 2 highlights the effects of vitamin D supplementation observed for the asthma-related outcome measures and for changes in serum 25(O)HD concentration. Only one study collected information on the number of emergency visits by group during treatment period. [42] Five studies compared the number of asthma exacerbations in children taking vitamin D3 supplementation versus control. [24,31,34,42,43] Six studies looked at asthma symptom scores [24,27,31,36,39,43] and one study reported on the change in the level of severity of asthma according to GINA guidelines. [42] One study reported on β2-agonist use. [39] Four studies compared the change in the daily steroid usage. [36,39,42,43] Seven studies examined the effect of vitamin D3 supplementation on FEV1 and/or PEF as measures of lung function. [24,27,31,36,39,42,43] Six studies reported on serum 25(OH)D levels at baseline and at follow-up within treatment groups.[24,27,31,39,42,43] No studies reported on days lost from work/school and nocturnal awakening. Two trials reported on adverse events. [34,43]

**Table 2:** Reported asthma-related outcomes of included trials.

	Outcomes*							
Study	ED visits for asthma	Asthma Control				Lung function		Serum 25(OH)D
		Exacerbations^‡^	Severity /symptoms^†^	Steroids use	β-2 agonist use	FEV1	PEF	
Baris et al, [43]	-	NS	NS	NS	-	NS	NS	**↑**
Yadav et al, [42]	**↓**	**↓**	**↓**	**↓**	-	-	**↑**	-
Darabi et al, [24]	-	**↓**	NS	-	-	NS	-	NS
Lewis et al, [27]	-	-	NS	-	-	NS	-	NS
Majak et al, [31]	-	**↓**	NS	-	-	NS	-	NS
Urashima et al, [34]	-	**↓**	-	-	-	-	-	-
Majak et al, [36]	-	-	NS	NS	-	NS	-	**↑**
Schou et al, [39]	-	-	NS	NS	NS	NS	NS	**↑**

Abbreviations: NS, non-significant effect; FEV1, forced expiratory volume in 1 second; PEF, peak expiratory flow;

^**↑**^, intervention significantly increased outcome measure as compared to control (P<0.05);

^**↓**^ intervention significantly decreased outcome measure as compared to control (P<0.05).

*Shows effect direction between intervention and control at the end of follow-up.

^†^Asthma symptoms score based on different scoring systems.

^‡^As defined by asthma exacerbation attacks.

Outcome based on two different units of measurement: number of children experiencing asthma exacerbation and number of asthma exacerbation during treatment period.

#### ED visits and hospitalizations for asthma

Yadav and Mittal reported a statistically significant reduction in the mean number of emergency visits in the vitamin D versus the placebo group (P = 0.015) when comparing the groups at monthly intervals up to the end of the study at 6 months. [42] However, none of the patients in either group had an ED visit after 3 months of treatment. None of the studies examined hospitalizations for asthma.

#### Asthma exacerbations

**Fig 2:**
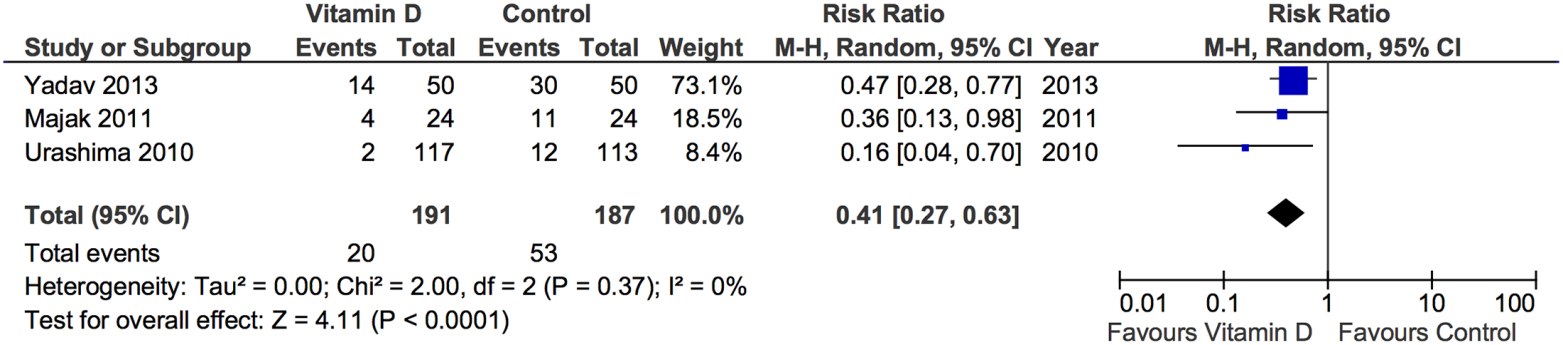
Forest plot of asthma exacerbations.

Asthma exacerbations were assessed in five trials, four of which that found that vitamin D supplementation decreased asthma exacerbations, [24,31,34,42] and one that reported no significant effect of the intervention (Table 2). Data was not available for the meta-analysis in two trials. [24,43] The number of children who experienced asthma exacerbation during the intervention was reported as a secondary outcome measure in five trials. [24,31,34,42,43] Of those, one trial defined asthma exacerbation as preceded by symptoms of an acute respiratory infection and requiring β2-agonists and antibiotics (if appropriate), [31] the second as physician-diagnosed asthma attack that included wheezing improved by inhalation of a β stimulant, [34] and three did not specify outcome ascertainment. [24,42,43] The pooled effect estimate for the trials by Majak et al (2011), [31] Urashima et al, [34] and Yadav and Mittal [42] was 0.41, (RR, 95% CI 0.27 to 0.63, P<0.0001) and no statistical heterogeneity was observed (*I* ^*2*^ = 0%, P = 0.37) (Fig 2).

#### Asthma severity/symptom scores

Trials that reported on asthma symptoms used different scoring systems. There was no effect of vitamin D supplementation on asthma symptom scores in the pooled analysis of the three trials that reported scores at baseline and follow-up using SMD (S1 Fig). [27,31,36] However, two studies noted a statistically significant improvement in asthma severity/asthma symptoms in favor of vitamin D group at six-month follow-up (P = 0.016 and P = 0.01). [42,43] Yadav and Mittal [42] recorded the change in asthma severity (mild, moderate persistent, severe persistent) based on the GINA (Global Initiative for Asthma) guidelines, [45] whereas Baris et al [43] used the total asthma symptom score [TASS] based on asthma symptoms recorded in daily diaries (zero point stands for no symptoms, 1 for mild, 2 for moderate, and 3 for severe). As Table 2 shows, no between-group effect of vitamin D supplementation was found on asthma symptoms scores at the end of follow-up using different assessment tools. Schou et al reported no effect but did not specify how asthma symptom scores were calculated. [39]

#### Lung function

Lung function was reported as FEV1% predicted, FEV1 (volume), or as peak expiratory flow rate (PEFR). Only one study reported a significant effect of vitamin D supplementation on lung function (Table 2). [42] For the purpose of the pooled analysis, we were unable to include data from three trials that did not report on FEV1% values. [24,39,42] For one trial, we obtained this data directly from the authors. [27] The pooled analysis of the results from the four trials presenting data on FEV1% is shown in the supporting information files (S2 Fig). The mean change from baseline in the intervention compared with the control group was null (0%, 95% CI, −3.17% to 3.18%, P = 0.99). There was no statistical heterogeneity for this estimate (*I* ^*2*^ = 0%, P = 0.66). Two studies reported the difference in mean PEFR across groups at the end of the intervention: Yadav and Mittal [42] reported a statistically significant improvement in the change in mean PEFR in the treatment arm as compared to the control (P<0.001) while Schou et al [39] reported no statistically significant differences between vitamin D and placebo periods. Baris et al reported baseline and follow-up values of peak expiratory flow as the percentage of predicted values for gender, age and height and found no effect of subcutaneous allergen immunotherapy treatment with vitamin D. [43]

#### *β*_2_-agonist use

In the crossover trial by Schou et al, there was no statistical difference found between vitamin D and placebo periods in the number of puffs of *β*_*2*_-agonist per day (P = 0.92). [39]

#### Steroid use

Of the four trials that reported on the change in inhaled steroid usage during the treatment period, three reported no effects of vitamin D supplementation [36,39,43] and one reported improvements in favour of the vitamin D group (P = 0.013). [42] However, the study by Baris et al reported that the rate of inhaled corticosteroids discontinuation was higher in the subcutaneous allergen immunotherapy plus vitamin D group as compared to that of immunotherapy alone (P = 0.02). [43]

#### Serum 25(OH)D concentration

Three trials reported a significant increase in mean serum 25(OH)D concentration in the vitamin D group as compared to the control group (Table 2). [36,39,43] Based on data from five trials that reported on the mean serum 25(OH)D concentration at the end of the vitamin D supplementation period, [27,31,36,39,43] we generated a pooled estimate for the mean difference between the vitamin D and the control group at the end of the supplementation period of 19.66 nmol/L (95% CI 5.96 to 33.37, P = 0.005; *I* ^*2*^ = 94%) (S3 Fig). We also performed a pooled analysis for the *change* in serum 25(OH)D concentration for the four trials that reported mean concentrations in each treatment arm at baseline and at the end of the vitamin D supplementation period. [27,31,36,43] For these four trials, the change in mean serum 25(OH)D concentrations at the end of the supplementation period compared to baseline differed by 2.41 nmol/L (95% CI -0.35 to 5.17, P = 0.09; *I* ^*2*^ = 57%)) between the vitamin D and the control group (S4 Fig).

#### Adverse effects

One trial reported no adverse events, [34] and one reported that levels of serum calcium, phosphorus, and parathyroid hormone as well as urinary calcium levels were within normal ranges in the vitamin D-receiving group. [43] Other trials did not mention adverse events.

### Quality of evidence

We found moderate-quality evidence for ED visits following asthma exacerbations and low-quality evidence for asthma exacerbation, asthma symptom scores, lung function (FEV1%, PEFR), and serum 25(OH)D, using the GRADE guidelines (Table 3). [46] Low-quality evidence according to the GRADE framework for quality assessment refers to evidence from RCTs with serious flaws for which any estimate of effect is uncertain. Reasons for rating down the quality of the body of evidence included uncertainty of allocation concealment and blinding (patients and outcome assessors), high rates of loss to follow-up (i.e., 20% or more), unclear reporting of outcome measurement, and suspicions of carryover effects due to inadequate wash-out period (crossover trial) (Table 3 and S3 Table). Asthma symptoms scores could not be compared directly due to differences in assessment methods and were therefore rated down. [18] The quality of evidence for the serum 25(OH)D was also rated down due to the inconsistency of results (significant heterogeneity) observed in the pooled analysis. Finally, we created funnel plots for the secondary outcomes (Figures A, B and C in S1 File), which showed some evidence of publication bias and suggested that smaller studies exaggerated intervention effect estimates. However, formal statistical tests for funnel plot asymmetry were not statistically significant (see S1 File).

**Table 3:** Quality assessment (GRADE evidence profile).

No. studies	Design	Risk of bias	Inconsistency	Indirectness	Imprecision	Publication bias	Intervention group (n)	Control group (n)	Effect	Quality	Importance
**Emergency department visits for asthma**											
1	RCT	No serious limitations	n/a	No serious indirectness	No serious imprecision	n/a	50	50	P = 0.015^g^	Moderate	Critical
**Asthma exacerbations (outcomes are number of participants experiencing asthma attacks and wheezing requiring beta2-agonists, use of beta2-agonists (puffs/day), and undefined asthma exacerbation attack)**											
6	RCT	Serious limitations^b^	No serious inconsistency	Serious indirectness^c^	No serious imprecision	None	257	250	0.41 (0.27 to 0.63)^f^; P<0.05^g^; no effect^d^	Low	Critical
**Asthma symptoms (outcomes are scores (in points) based on ACT, ATAQ for children, daily diary card, ACQ and undefined asthma symptom scores)**											
6	RCT	Serious limitations^a^	No serious inconsistency	Serious indirectness^c^	No serious imprecision	None	117	114	No effect^d^; P = 0.01(6mo follow-up)^g^	Low	Critical
**Lung function (outcomes are FEV1 (L in 1 sec or % of predicted value) and PEF (mL/min)**											
7	RCT	Serious limitations^a^	No serious inconsistency	No serious indirectness	No serious imprecision	None	167	164	0.00 (-3.17 to 3.18)^e^; P<0.001^g^; no effect^d^	Low	Critical
**Serum 25(OH)D (nmol/L)**											
6	RCT	Serious limitations^a^	Serious inconsistency^h^	No serious indirectness	No serious imprecision	None	117	114	19.66 (5.96 to 33.37)^i^; no effect^d^	Low	Important

Abbreviations: ACT, Asthma Control Test; ATAQ, Asthma Therapy Assessment Questionnaire; ACQ, Asthma Control Questionnaire; FEV1, forced expiratory volume in 1 second; PEF, peak expiratory flow rate.

^a^Unclear allocation concealment, blinding of participants and outcome assessors, accounting of patients and outcome events, and other risk of bias (carryover effects in crossover trial).

^b^Unclear allocation concealment, blinding of participants and outcome assessors, accounting of patients and outcome events.

^c^Differences in interventions and outcomes measured across studies.

^d^Non-significant effect across studies not included in the meta-analysis.

^e^Weighted difference in mean (WMD) change between intervention and control group.

^f^Risk ratio (RR): risk of experiencing asthma exacerbation in the intervention group as compared to the control group.

^g^Not included in the meta-analysis; favours intervention group.

^h^Significant statistical heterogeneity observed based on random effects meta-analysis.

^i^Weighted mean difference (WMD) at end of intervention between intervention and control group.

## Discussion

Our systematic review and meta-analysis provides weak evidence to support vitamin D supplementation for the reduction of asthma exacerbations. Among the studies that found reduced exacerbations, changes in serum 25(OH)D concentrations after supplementation were either not significant or not reported, which further weakens the causal interpretation of the pooled estimate of effect reported on this outcome. The evidence for other asthma-related outcomes is either limited or inconclusive, with the majority of trials demonstrating statistically non-significant improvements in asthma symptoms, lung function, and β_2_-agonist use and daily steroid use.

Two systematic reviews and meta-analyses of randomized controlled trials in children with asthma published recently yielded similar results. [47,48] However, the current review synthesizes the literature from more recently published trials not included in previous reviews. Compared to the review by Fares et al., three RCTs were published since their search was completed, [24,42,43] and we included an additional RCT published in 2010 not included in their review. [34] While Fares et al. could not conduct a meta-analysis with only one included study reporting on asthma exacerbations, we reviewed this outcome in six studies and conducted the meta-analysis using three studies. The review by Pojsupap et al. [48] focused on high supplemental dose of vitamin D (500IU/day or more) and included a total of five trials while we included two additional trials with doses of 500IU/day or more, [24] and one trial with lower doses. [36] We reported the same estimate of effect on asthma exacerbations; however, we considered the quality of evidence to be low (outcome ascertainment unclear in one trial and no published protocol for two trials). Further, we are the only review to report pooled quantitative estimates of effect on pulmonary function (FEV1) based on four trials and 134 participants, including data obtained directly from authors in one trial. [27]

Our findings are also consistent with other reviews of observational studies looking at vitamin D and asthma in children. [49–52] The review of observational studies by Gupta et al reported a cross-sectional association between low serum levels of vitamin D in children and mild to moderate asthma, reduced lung function, reduced asthma control and more asthma exacerbations. [50] The potential for confounding by the environment, race/ethnicity, diet, atopy, and concurrent treatment in observational studies limit our ability to identify a causal effect of vitamin D. [53]For example, it is possible that children with asthma and poorer lung function spend more time indoors, thus having decreased vitamin D synthesis via sun exposure on the skin, as well as increased indoor environmental allergen exposures.

Several studies have provided non-experimental evidence supporting the inverse relationship between vitamin D levels and asthma exacerbations. [35,54,55] Likely mechanisms by which adequate vitamin D levels may decrease the risk of asthma exacerbations have been proposed, including better handling of respiratory infections, [56] enhanced anti-inflammatory functions of corticosteroids in asthma, [56–59] and through reduced airway inflammation and airways obstruction. [56,60,61] A large RCT in adults with asthma and low vitamin D levels (n = 408) found that vitamin D3 supplementation as an adjunct therapy to inhaled corticosteroids resulted in non-significant reductions in the rate of first asthma exacerbation (adjusted HR 0.7; 95% CI 0.4 to 1.2]; *P* = 0.21), and in the overall exacerbation rate (adjusted HR 0.63; 95% CI, 0.39 to 1.01; P = 0.05). [62] The authors noted that the failure to observe a statistically significant effect could be attributed to inadequate power and a lower than expected event rate in the control group. The overall evidence for reducing asthma exacerbations in the current review as well as other published experimental and non-experimental studies lend some support to vitamin D supplementation as a potential target to aid in asthma management.

### Strengths and limitations

We aimed to minimize bias by applying systematic methods to the process of selecting, synthesizing, and assessing the published evidence. The diversity in patient population and intervention of included trials contribute some evidence of external validity the results presented in this review.

The current review has methodological limitations that should be taken into account when interpreting the results. At the study level, it was impossible to fully assess the risk of bias for the majority of the trials largely due to incomplete reporting of the methods for allocation concealment or blinding. At the outcome level, only a small number of trials contributed data to the meta-analyses. The estimate of effect would have been strengthened if data had been available for standardized outcomes and if comparable methods of measurement or ascertainment had been used across studies (e.g. asthma exacerbations and asthma symptoms). Further, there was clinical diversity in the patient population and interventions. While race/ethnicity likely affects vitamin D concentrations in response to supplementation, [63] we could not account for this in our meta-analysis because this information was not reported in most trials. For the interventions, the vitamin D regimen varied by type and duration. The smaller doses given in some trials might have been insufficient to elucidate effects, where the change from baseline to follow-up in serum 25 (OH)D did not differ between the intervention and control groups. We explored this by plotting standardized effect sizes across included trials by the daily dose of vitamin D supplementation (S5 Fig). Our exploratory analysis revealed that inconsistencies of effects tend to occur at lower doses (< 500 IU/d).

The small sample sizes of trials included in pooled analyses resulted in poor precision of effect estimates and prevented us from conducting sensitivity. Further, because vitamin D is thought to enhance the response to steroids and to play a role in the regulation of immune function, it would have been relevant to perform sub-group analyses on trials in which vitamin D was given as an adjunct to improve the clinical efficacy of other forms of asthma treatment. However, we could not perform such analyses and the effect estimates obtained from combining trials with vitamin D given alone or as an adjunct (to steroids and/or immunotherapy) may mask this biological interaction effect. In the absence of a sufficient number of trials that would allow direct comparisons of similar interventions on standardized and clinically important outcomes, our ability to detect a causal effect for a defined intervention was limited.

## Conclusion

RCTs provide limited evidence for a reduction in asthma exacerbations but larger trials are necessary. The current review found moderate evidence based on one study that monthly doses of 60,000 IU of vitamin D may help in preventing ED visits, although more studies are needed to corroborate this finding. The wide range of doses given across studies reflects the lack of consensus on the optimal level of total serum 25(OH)D to elucidate benefits on non-skeletal outcomes. To best inform guidelines on vitamin D supplementation in children with asthma, future trials should investigate primarily on standardized and patient-relevant outcomes that are comparable across studies. These may include standardized definitions of asthma exacerbations, and important health utilization outcomes such as ED visits and hospitalizations for asthma. Currently, several unpublished trials on the effect of vitamin D supplementation on asthma outcomes in children are in progress. Future meta-analyses should examine separately the potential for vitamin D supplementation as an adjunct to other forms of asthma treatment.

## Supporting Information

S1 Fig Forest plot of change in asthma symptom scores. (TIFF)

S2 Fig Forest plot of change in FEV1%. (TIFF)

S3 Fig Forest plot of final serum 25(OH)D concentration. (TIFF)

S4 Fig Forest plot of change in serum 25(OH)D concentration. (TIFF)

S5 Fig Exploratory Analysis. (TIFF)

S1 File Funnel plots. (DOCX)

S1 Table Search strategy. (DOCX)

S2 Table PRISMA checklist. (DOCX)

S3 Table Study Limitations and Risk of Bias. (DOCX)
