# Supplementary figures and images for: Vitamin D Supplementation for Childhood Asthma: A Systematic Review and Meta-Analysis
Source: PLoS One. 2015 Aug 31;10(8):e0136841. doi: 10.1371/journal.pone.0136841 (PMC4556456; doi:10.1371/journal.pone.0136841)

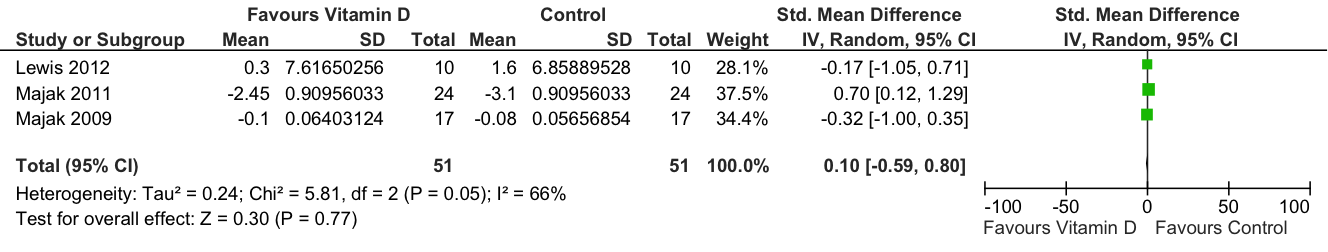

Supplement: S1 Fig — (TIFF) [file pone.0136841.s001.tiff]

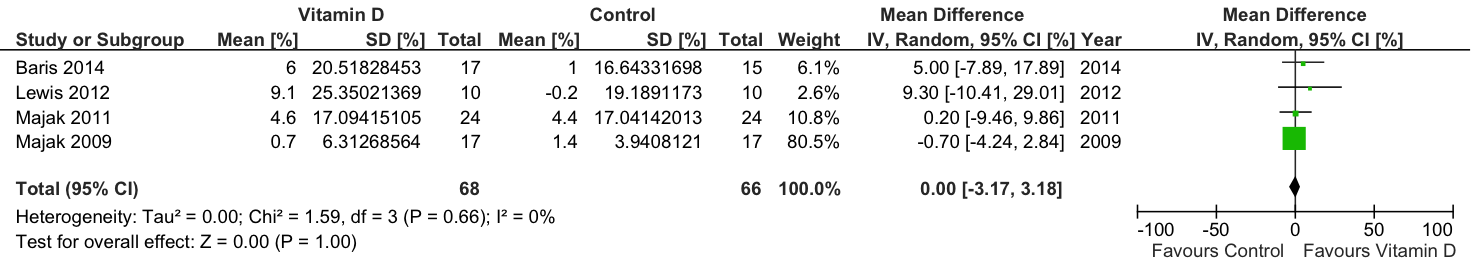

Supplement: S2 Fig — (TIFF) [file pone.0136841.s002.tiff]

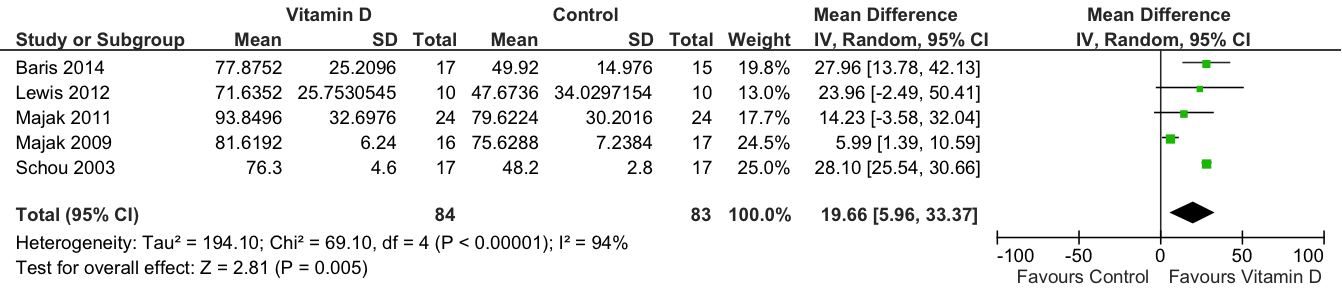

Supplement: S3 Fig — (TIFF) [file pone.0136841.s003.tiff]

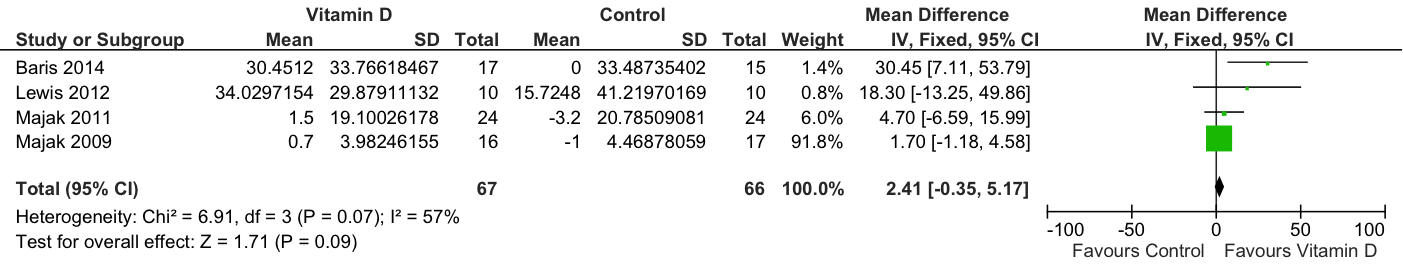

Supplement: S4 Fig — (TIFF) [file pone.0136841.s004.tiff]

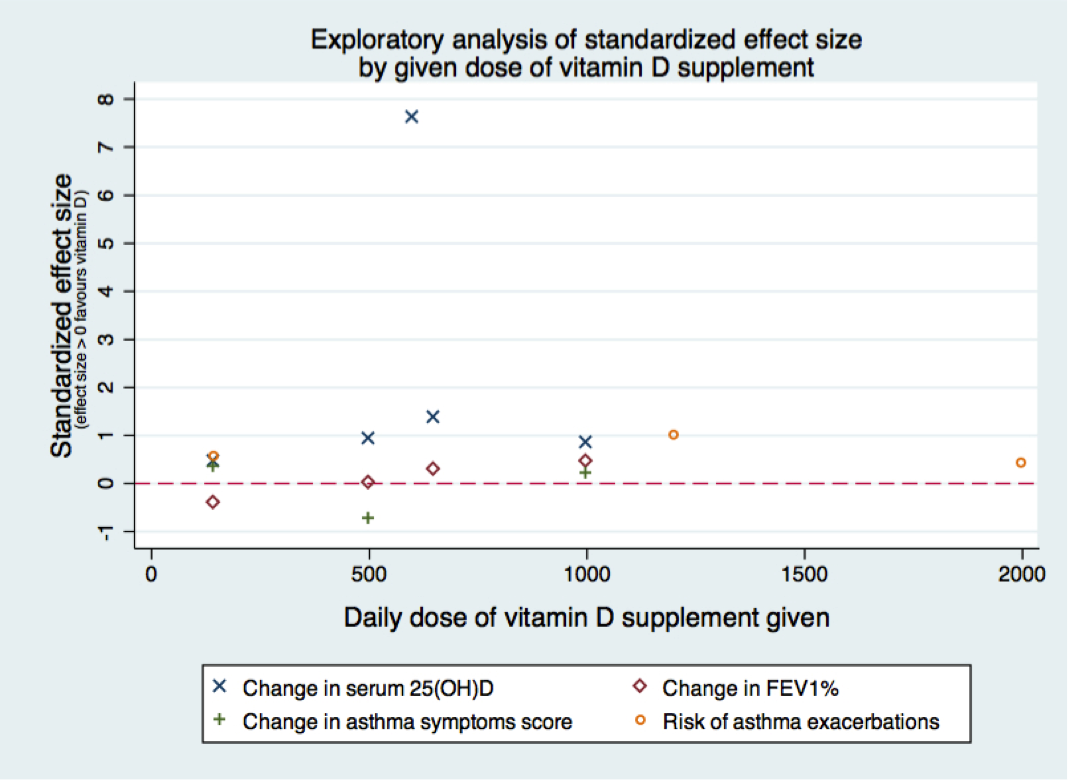

Supplement: S5 Fig — (TIFF) [file pone.0136841.s005.tiff]
