# Supplementary material for: Vitamin D Supplementation for Childhood Asthma: A Systematic Review and Meta-Analysis
Source: PLoS One. 2015 Aug 31;10(8):e0136841. doi: 10.1371/journal.pone.0136841 (PMC4556456; doi:10.1371/journal.pone.0136841)
Supplement: S1 File — (DOCX) [file pone.0136841.s006.docx]

**S1 File. Funnel plots for detecting bias in meta-analyzed outcomes**

**Figure A. Asthma exacerbations**

**
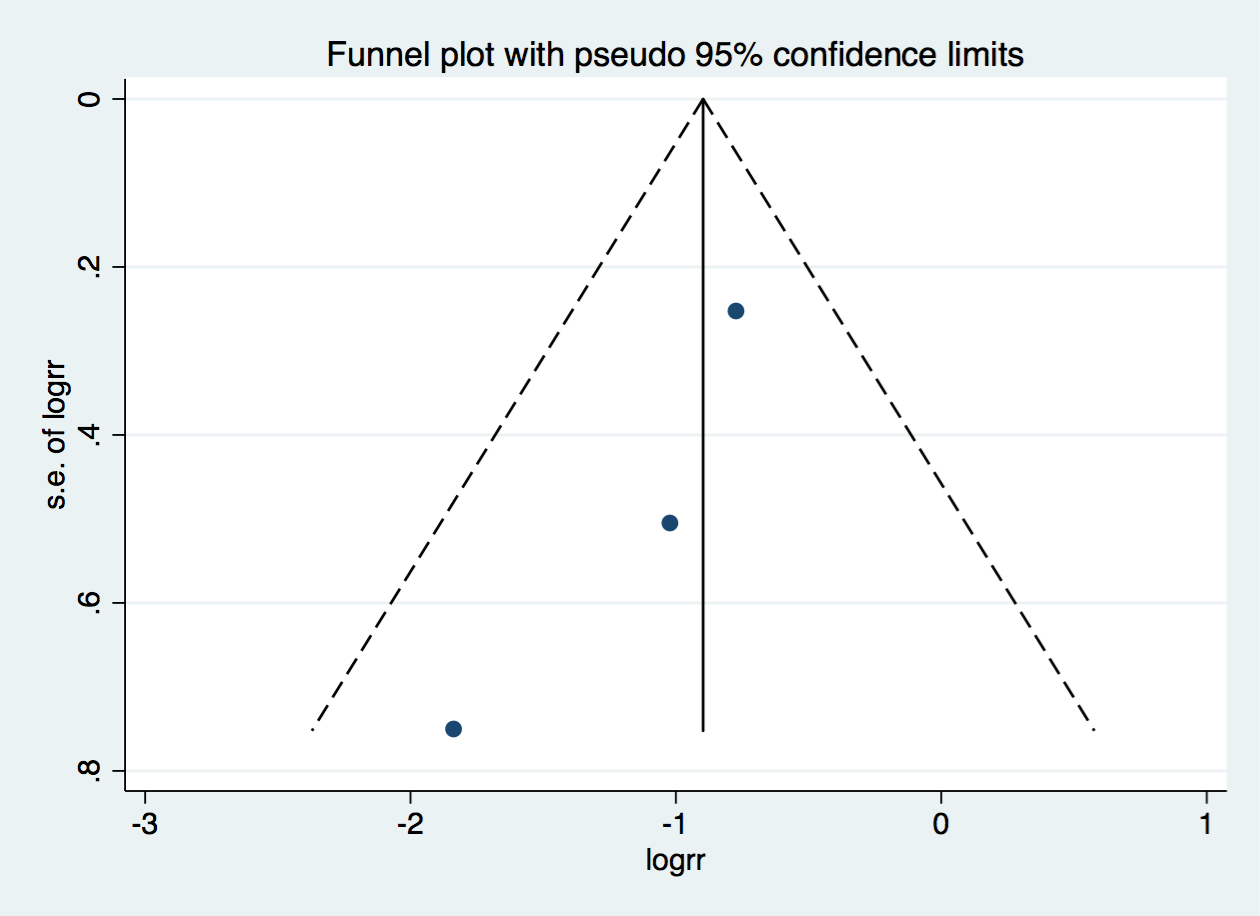
**

Begg's test for small-study effects:P = 0.296 (continuity corrected)

**Figure B. Change in asthma symptoms**


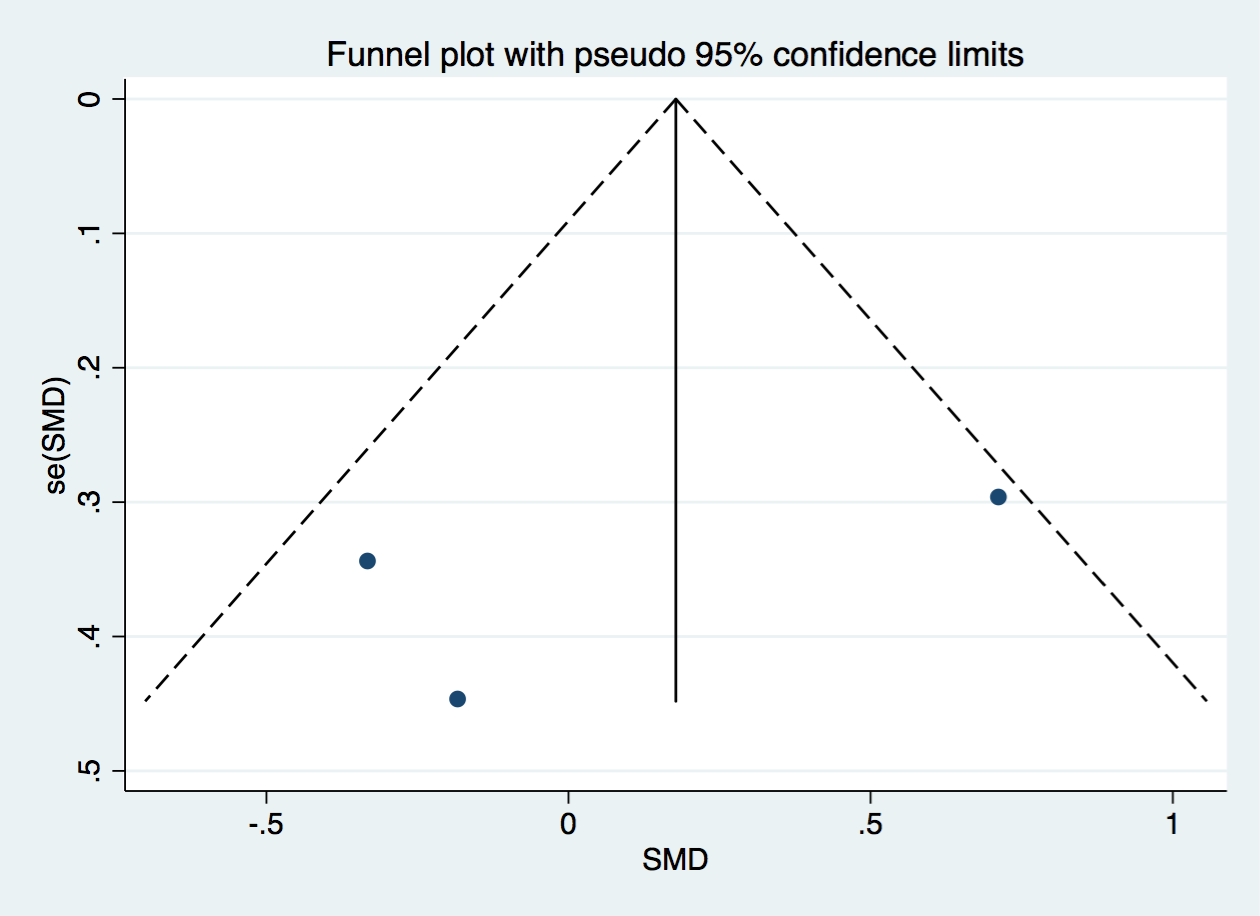


Egger's test for small-study effects: P = 0.521

**Figure C. Change in FEV1%**


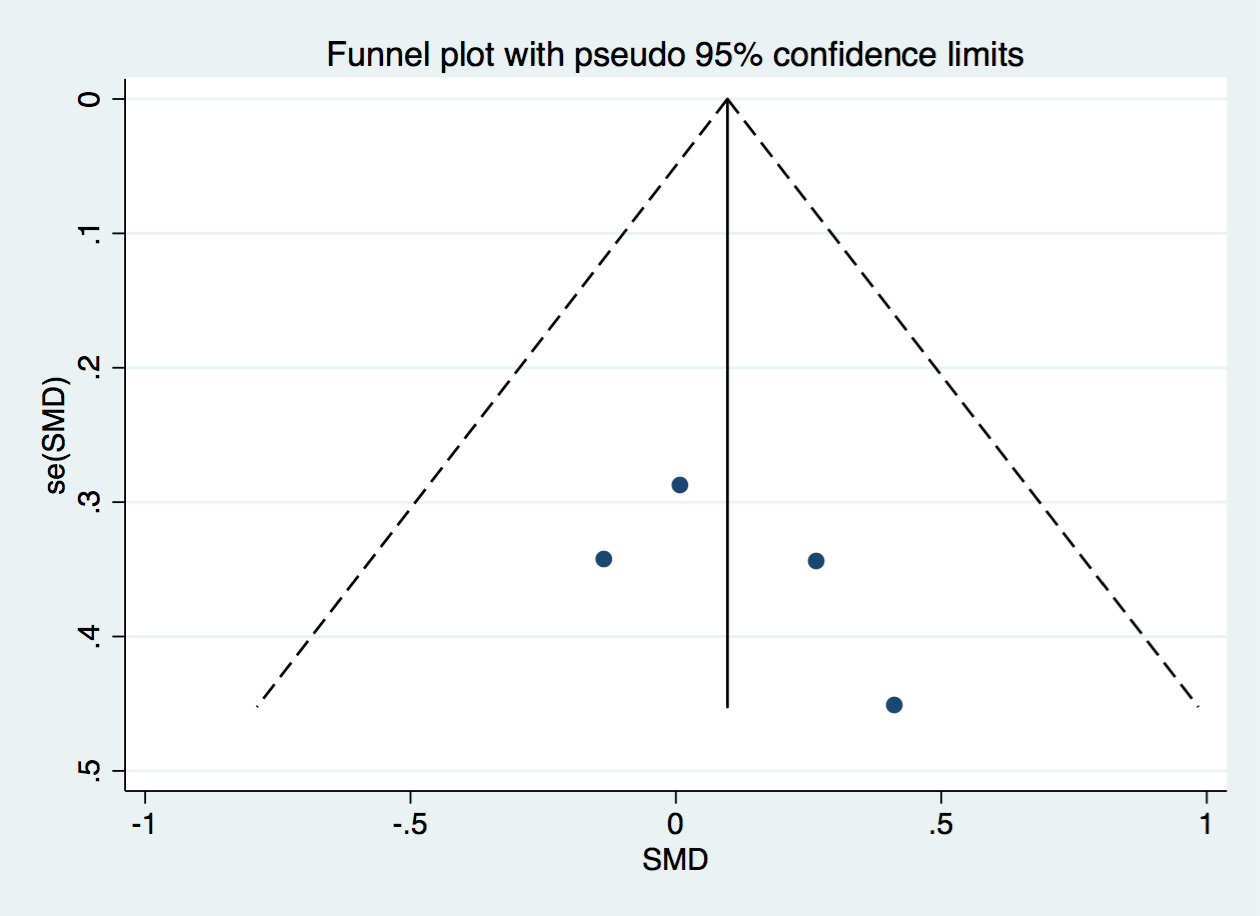


Egger's test for small-study effects: P = 0.344
