# Supplementary material for: Vitamin D Supplementation for Childhood Asthma: A Systematic Review and Meta-Analysis
Source: PLoS One. 2015 Aug 31;10(8):e0136841. doi: 10.1371/journal.pone.0136841 (PMC4556456; doi:10.1371/journal.pone.0136841)
Supplement: S1 Table — (DOCX) [file pone.0136841.s007.docx]

**S1.0 Search strategy**

| **MEDLINE (1946-July 2013)** | **Embase (1947-July 2013)** | **CENTRAL (1991-July 2013)** | **CINHAL** |
| --- | --- | --- | --- |
| 1. exp Vitamin D/ | 1. exp Vitamin D/ | #1. MeSH descriptor: [Vitamin D] explode all trees | ((MH Child) OR (MH Adolescence)) AND ((((MH Vitamin D) OR (MM Ergocalciferols) OR (MM Cholecalciferol) OR (MM Calcitriol)) AND ((TI (vitamin d OR vitamin d2 OR vitamin d3 OR 25-hydroxyvitamin OR ergocalciferol OR colecalciferol OR cholecalciferol OR calcitriol OR hydroxyvitamin d) OR AB (vitamin d OR vitamin d2 OR vitamin d3 OR 25-hydroxyvitamin OR ergocalciferol OR colecalciferol OR cholecalciferol OR calcitriol OR hydroxyvitamin d)) OR ((MH Vitamin D) OR (MM Ergocalciferols) OR (MM Cholecalciferol) OR (MM Calcitriol)))) AND (((MH Asthma) OR (MM Asthma Exercise-Induced)) AND ((TI (asthma OR asthmatic OR wheeze OR wheezing OR reactive airway OR respiratory hypersensitivity OR bronchial hyperreactivity) OR AB (asthma OR asthmatic OR wheeze OR wheezing OR reactive airway OR respiratory hypersensitivity OR bronchial hyperreactivity)) OR ((MH Asthma) OR (MM Asthma Exercise-Induced)))) AND ((MH Child) OR (MH Adolescence))) |
| 2. exp Hydroxycholecalciferols/ | 2. exp Hydroxycholecalciferols/ |  |  |
| 3. exp Cholecalciferol/ | 3. exp Colecalciferol/ | #2. MeSH descriptor: [Ergocalciferol] explode all trees |  |
| 4. exp Ergocalciferol/ | 4. exp Ergocalciferol/ |  |  |
| 5. exp dihydrotachysterol/ | 5. exp dihydrotachysterol/ | #3. Vitamin D:ti,ab |  |
| 6. exp 25-hydroxyvitamin d2/ | 6. exp 25-hydroxyvitamin d2/ | #4. cholecalciferol*:ti,ab |  |
| 7. alfacalcidol$.mp. | 7. alfacalcidol$.mp. | #5. calcifediol*:ti,ab |  |
| 8. alphacalcidol$.mp. | 8. alphacalcidol$.mp. | #6. calcitriol*:ti,ab |  |
| 9. colecalciferol$.mp. | 9. colecalciferol$.mp. | #7. dihydrotachysterol*:ti,ab |  |
| 10. cholecalciferol$.mp. | 10. cholecalciferol$.mp. | #8. *hydroxyvitamin* d*:ti,ab |  |
| 11. calcifediol$.mp. | 11. calcifediol$.mp. | #9. #1 or #2 or #3 or #4 or #5 or #6 or #7 or #8 |  |
| 12. calcitriol$.mp. | 12. calcitriol$.mp. |  |  |
| 13. dihydrotachysterol$.mp. | 13. dihydrotachysterol$.mp. | #5 MeSH descriptor: [Child] explode all trees |  |
| 14. hydroxyvitamin$.mp. | 14. hydroxyvitamin d$.mp. |  |  |
| 15. ergocalciferol.mp. | 15. ergocalciferol.mp. | #6 MeSH descriptor: [Infant] explode all trees |  |
| 16. vitamin$ adj2 (d or d2 or d3).ti,ab | 16. vitamin$ adj2 (d or d2 or d3).ti,ab |  |  |
| 17. (“vitamin d” or “vitamin d2” or “vitamin d3”).ti,ab | 17. (“vitamin d” or “vitamin d2” or “vitamin d3”).ti,ab | #7 MeSH descriptor: [Adolescent] explode all trees |  |
| 18. or/1-17 | 18. or/1-17 |  |  |
| 19. asthma$.mp. | 19. asthma$.mp. | #8. MeSH descriptor: [Pediatrics] explode all trees |  |
| 20. reactive airway$.mp. | 20. reactive airway$.mp. |  |  |
| 21. wheez$.mp. | 21. wheez$.mp. | #9. #5 or #6 or #7 or #8 |  |
| 22. exp Asthma/ | 22. exp asthma/ | #10. MeSH descriptor: [Asthma] explode all trees |  |
| 23. exp Respiratory Hypersensitivity/ | 23. exp respiratory hypersensitivity/ |  |  |
| 24. exp Bronchial Hyperreactivity/ | 24. exp bronchial hyperreactivity/ | #11. #4 and #9 and #10 |  |
| 25. or/19-24 | 25. or/19-24 |  |  |
| 26. exp child/ | 26. exp child/ |  |  |
| 27. child$.mp. | 27. child$.mp. |  |  |
| 28. exp pediatrics/ | 28. exp pediatrics/ |  |  |
| 29. pediatric$.mp. | 29. pediatric$.mp. |  |  |
| 30. paediatric$.mp. | 30. paediatric$.mp. |  |  |
| 31. or/26-30 | 31. or/26-30 |  |  |
| 32. premature*.mp. | 32. premature*.mp. |  |  |
| 33. preterm*.mp. | 33. preterm*.mp. |  |  |
| 34. perinat$.mp. | 34. perinat$.mp. |  |  |
| 35. neonat$.mp. | 35. neonat$.mp. |  |  |
| 36. newborn$.mp. | 36. newborn$.mp. |  |  |
| 37. new born$.mp. | 37. new born$.mp. |  |  |
| 38. infan$.mp. | 38. infan$.mp. |  |  |
| 39. bab$.mp. | 39. bab$.mp. |  |  |
| 40. toddler$.mp. | 40. toddler$.mp. |  |  |
| 41. boy$.mp. | 41. boy$.mp. |  |  |
| 42. girl$.mp. | 42. girl$.mp. |  |  |
| 43. kid$1.mp. | 43. kid$1.mp. |  |  |
| 44. school$.mp. | 44. school$.mp. |  |  |
| 45. juvenil$.mp. | 45. juvenil$.mp. |  |  |
| 46. underage$.mp. | 46. underage$.mp. |  |  |
| 47. under age$.mp. | 47. under age$.mp. |  |  |
| 48. teen$.mp. | 48. teen$.mp. |  |  |
| 49. minor$.mp. | 49. minor$.mp. |  |  |
| 50. youth$.mp. | 50. youth$.mp. |  |  |
| 51. pubescen$.mp. | 51. pubescen$.mp. |  |  |
| 52. adolescen$.mp. | 52. adolescen$.mp. |  |  |
| 53. or/32-52 | 53. or/32-52 |  |  |
| 54. infan$.jw. | 54. infan$.jw. |  |  |
| 55. child$.jw. | 55. child$.jw. |  |  |
| 56. pediatric$.jw. | 56. pediatric$.jw. |  |  |
| 57. paediatric$.jw. | 57. paediatric$.jw. |  |  |
| 58. adolescen$.jw. | 58. adolescen$.jw. |  |  |
| 59. or/54-58 | 59. or/54-58 |  |  |
| 60. 31or 53 or 59 | 60. 31or 53 or 59 |  |  |
| 61. 18 and 25 and 60 | 61. 18 and 25 and 60 |  |  |
