# Supplementary material for: Vitamin D Supplementation for Childhood Asthma: A Systematic Review and Meta-Analysis
Source: PLoS One. 2015 Aug 31;10(8):e0136841. doi: 10.1371/journal.pone.0136841 (PMC4556456; doi:10.1371/journal.pone.0136841)
Supplement: S3 Table — (DOCX) [file pone.0136841.s009.docx]

**S4.0 Study Limitations and Risk of Bias (15)**

| **Study** | **Lack of allocation concealment** | **Lack of blinding** | **Incomplete accounting of patients and outcome events** | **Selective outcome reporting bias^b^** | **Other*** |
| --- | --- | --- | --- | --- | --- |
| Baris et al (43) | No major risk of bias | No major risk of bias | Major risk of bias | Unclear | No major risk of bias |
| Yadav et al (42) | No major risk of bias | No major risk of bias | No major risk of bias | Unclear | Major risk of bias^d^ |
| Darabi et al (24) | Unclear | Unclear | Major risk of bias^a^ | Unclear | Unclear |
| Lewis et al (27) | Unclear | Unclear | Major risk of bias^a^ | Unclear | No major risk of bias |
| Majak et al (31) | No major risk of bias | Unclear | No major risk of bias | Unclear | No major risk of bias |
| Urashima et al (34) | No major risk of bias | No major risk of bias | Major risk of bias^a,c^ | Unclear | No major risk of bias |
| Majak et al (36) | Unclear | No major risk of bias | No major risk of bias | Unclear | No major risk of bias |
| Schou et al (39) | No major risk of bias | Unclear | No major risk of bias | Unclear | Major risk of bias^e^ |
| *Includes the following: stopping early for benefit, use of unvalidated outcome measures (e.g., patient-reported outcomes), carryover effects in crossover trial, and recruitment bias in cluster-randomized trials. | | | | | |
| ^a^Includes the following: participants lost to follow-up > 20%, lack of assessment by intention-to-treat analysis in the presence of non-compliance and missing outcomes. | | | | | |
| ^b^Could not have access to a protocol developed before the study was undertaken. | | | | | |
| ^c^Recruitment was not based on asthma diagnosis; a subgroup of participants (230/430) with a previous diagnosis of asthma were used for secondary analyses. | | | | | |
| ^d^ Unclear outcome measures for asthma exacerbation.  ^e^ Carryover effects because of crossover design; insufficient wash-out period (2 weeks). | | | | | |
